# Bronchoscopic observation with linked colour imaging

**DOI:** 10.1002/rcr2.399

**Published:** 2019-02-14

**Authors:** Shinichi Yamamoto, Tomoki Shibano, Masaya Sogabe, Hideki Negishi, Sayaka Mitsuda, Shunsuke Endo

**Affiliations:** ^1^ Department of General Thoracic Surgery Jichi Medical University Shimotsuke Japan

**Keywords:** Bronchoscopy, inflammatory change, linked colour imaging, lung cancer

## Abstract

We report two cases of the comparison of diagnosis made with linked color imaging (LCI) and conventional white‐light imaging (WLI) on the same patients. In case 1, a 75‐year‐old man in whom right upper lobectomy with mediastinal lymph node dissection was performed due to lung cancer had signs of bronchitis on postoperative day 8. The LCI demonstrated slight inflammatory changes that were not detectable with the conventional WLI on the tracheal wall. In case 2, in a 61‐year‐old woman who was diagnosed with adenoid cystic carcinoma, the bronchial wall was checked to confirm the extent of the tumour. The submucosal vascularity and tumour margin on the bronchial mucosa were better visible on LCI than on WLI. We could easily detect the mucosal inflammatory lesion and the malignant lesion with LCI in comparison with conventional WLI. Both mucosal inflammatory and malignant lesions were better visible with LCI in comparison to WLI.

## Introduction

Bronchoscopy is a common and essential procedure for the detection of central‐airway benign and malignant lesions. In some cases, these lesions may be too thin or diminutive to be detected with conventional bronchoscopy.

Linked colour imaging (LCI) is a novel image‐enhanced endoscopy technology developed by the Fujifilm Corporation (Tokyo, Japan). LCI acquires images by simultaneously using narrow‐band short‐wavelength light and white light in an appropriate balance 1. This combination of light provides more information about the vasculature and architecture of the mucosal surface than that obtained with conventional white light imaging (WLI). The difference on the hue of the endoscopic images can be enhanced by unique image processing, which may make the red region redder and the white region whiter.

The technology is widely used in gastrointestinal endoscopy and colonoscopy for detecting gastric cancer, colon cancer, and inflammatory changes 1. Recently, the system of LCI for bronchoscopy was developed as the following equipment: LASEREO bronchoscopic system and bronchoscope EB‐580S (FUJIFILM Co., Tokyo, Japan). We can instantly switch from WLI to LCI using a button on the top of the bronchoscope handle. It is anticipated that LCI will be useful for diagnostic bronchoscopy, especially in detecting inflammatory changes and tracheobronchial carcinoma. However, there is no report of LCI for bronchoscopy.

Here, we report two cases of the comparison of diagnosis made with LCI and conventional WLI on the same patients.

## Case Report

### Case 1

**Figure 1:**
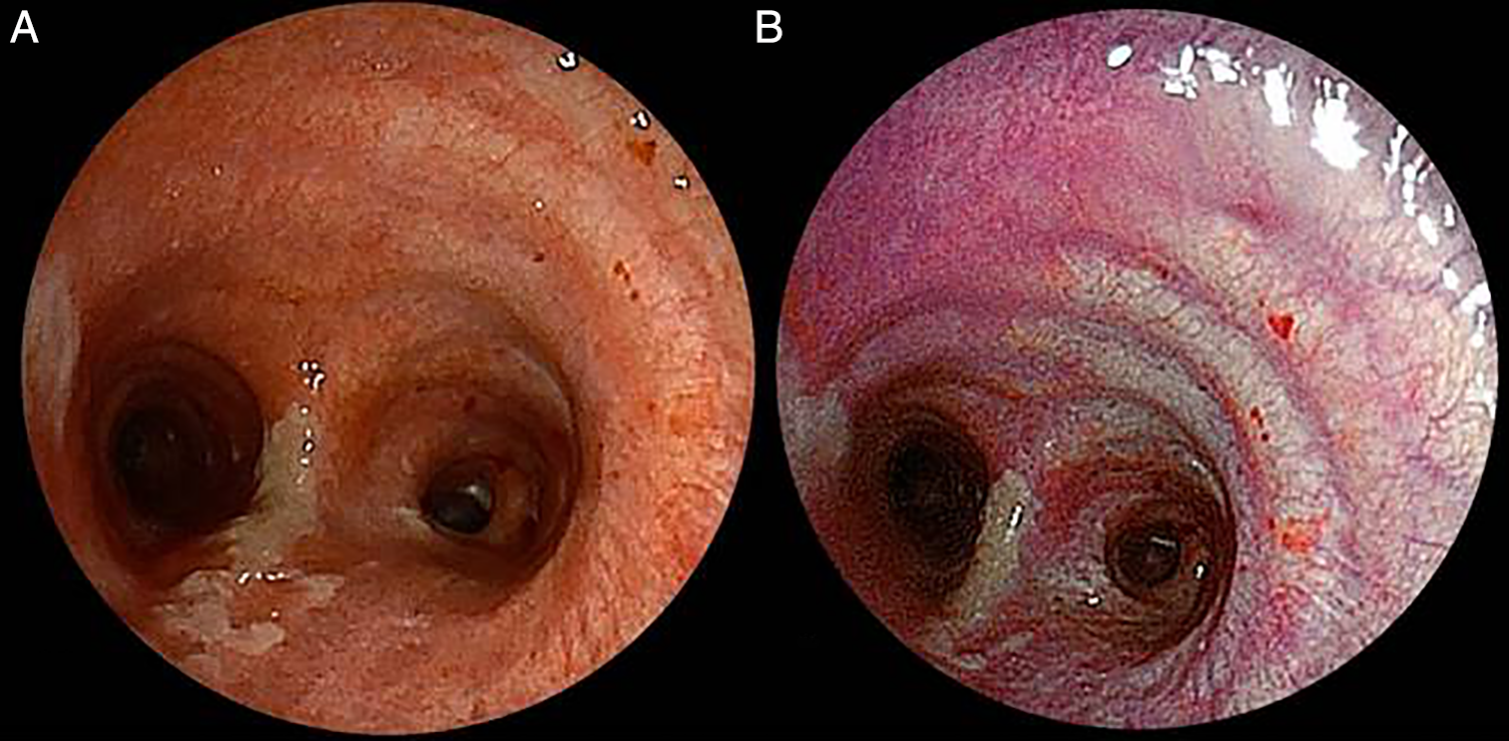
The case of postoperative bronchitis: linked colour imaging (LCI) clearly showed the difference of mucosal colour change between inflamed bronchial mucosa and normal bronchial mucosa. (A) White‐light imaging (WBI); (B) LCI.

In a 75‐year‐old man, right upper lobectomy with mediastinal lymph node dissection was performed due to lung cancer. He had yellow sputum expectoration on postoperative day 8, and the level of C‐reactive protein (CRP) was elevated in spite of no abnormal findings on chest X‐ray. Bronchoscopy was performed immediately to check the presence of postoperative bronchitis or bronchopleural fistula. Although purulent secretions were clearly visible on WLI, there were no signs of ischaemic bronchitis and bronchopleural fistula. The LCI demonstrated the contrast between bronchial mucosa and purulent secretions and detected inflammatory change that was not detectable with the conventional WLI on the tracheal wall (Fig. 1A, B). His sign improved and the level of CRP was normalized because of the administration of antibiotics.

### Case 2

**Figure 2:**
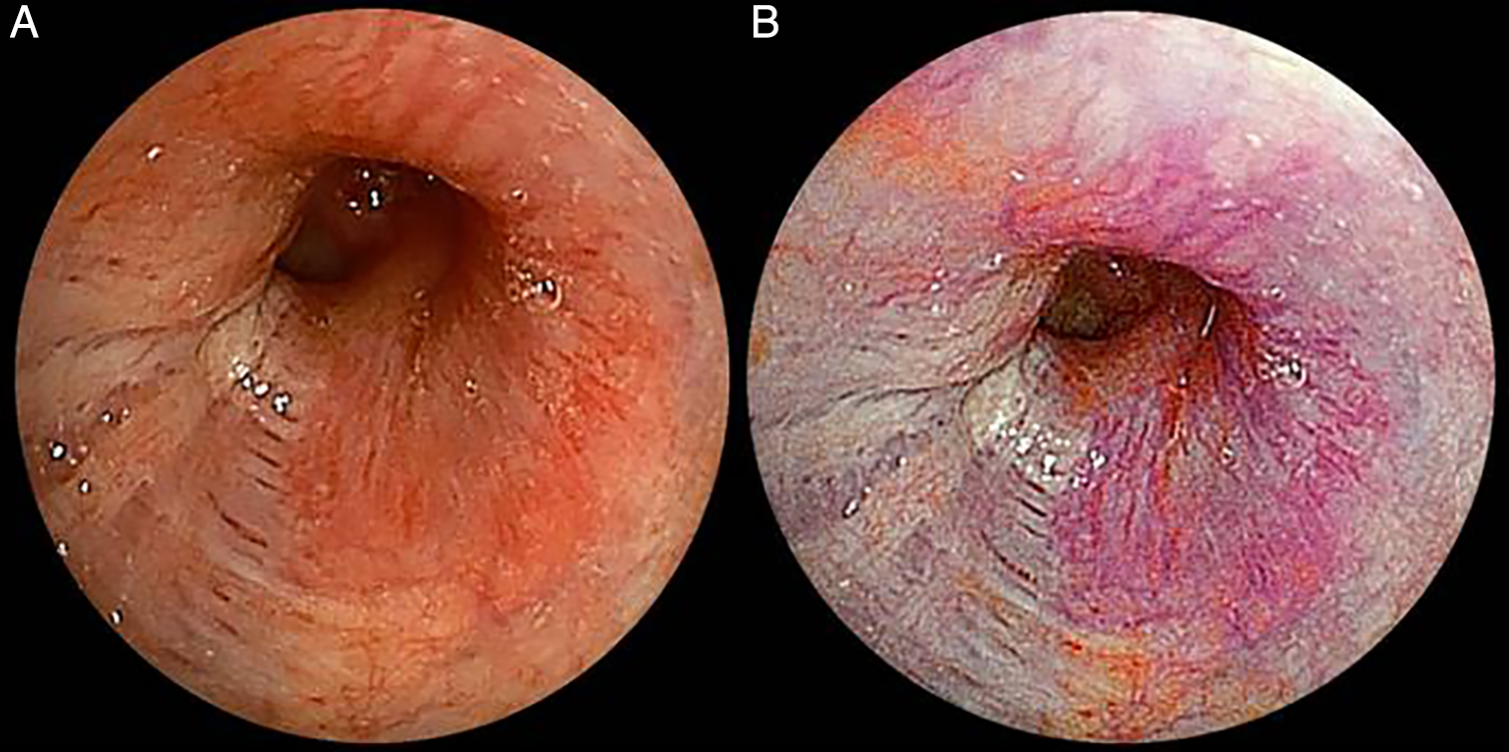
The case of adenoid cystic carcinoma in the left main bronchus: linked colour imaging (LCI) clearly showed the difference of submucosal vascularity and tumour margin on the bronchial mucosa. (A) White‐light imaging (WLI); (B) LCI.

A 61‐year old woman who was diagnosed with adenoid cystic carcinoma in her left main bronchus was admitted for surgical treatment. Bronchoscopy was performed to confirm the extent of the tumour. The submucosal vascularity and tumour margin on the bronchial mucosa were more clearly visible on LCI than on WLI (Fig. 2A, B). According to the findings, we could perform left pneumonectomy by clearly securing the surgical margin with LCI.

## Discussion

This is the first report on applying an LCI system to diagnostic bronchoscopy. We can clearly detect the extent of the lesion with LCI, which has a colour contrast between red and white.

Bronchoscopy has an important role to detect inflammatory changes on the bronchi, especially for postoperative lung cancer patients. Some patients have postoperative ischaemic bronchitis, which causes severe complications such as bronchopleural fistula and acute respiratory distress syndrome 2. In case 1, we detected postoperative bronchitis with LCI better than with WLI. The early detection of the inflammatory changes may distinguish bronchitis from progressive postoperative complications. In case 2, we could estimate the resection line of the bronchus before the surgery because of the LCI images. The determination of surgical margin for tracheobronchial malignant lesions was very important for thoracic surgeons in order to determine pneumonectomy.

Narrow‐band imaging (NBI) has been developed and widely used for the detection of subtle changes of bronchial mucosa, such as early bronchial malignant lesions, and also some benign diseases 3. However, NBI is criticized for darkness due to limited wavelength. Moreover, the interpretation of NBI findings requires special training because of the difference of colour balance. One of the qualities of LCI is that the brightness of images of LCI can be maintained because of the availability of WLI plus narrow‐band wavelength 4. Another characteristic of LCI is that the LCI images resemble conventional WLI compared to the images of NBI 5. NBI applies limited wavelengths using a narrow‐band imaging filter. On the other hand, the acquired colour information with LCI is reallocated to differentiate the colours close to the genuine mucosal colours compared to NBI. Therefore, oversights of the subtle bronchial mucosal lesions can be prevented because of the brightness and contrast even if less‐experienced bronchoscopists perform the evaluation of imagings.

There are some limitations to the study. First, LCI findings do not reflect the pathological differences because they show just submucosal colour changes. Comparing bronchoscopic findings and pathological findings is our next research task. Second, it is difficult to check the deeper layers of the submucosa because it does not impact the changes of mucosal colour. We need further studies to clarify the relationship between colour change and bronchial structure.

In conclusion, mucosal inflammatory lesions and vascularity of malignant lesions were more easily detected with LCI in comparison with conventional WLI. This system may facilitate the diagnosis of changes on the bronchial mucosa associated with bronchitis and lung cancer.

### Disclosure Statements

Appropriate written informed consent was obtained for publication of this case report and accompanying images. The authors of this study borrowed an LCI bronchoscope system from Fujifilm Corporation.
